# Sorting Signals, N-Terminal Modifications and Abundance of the Chloroplast Proteome

**DOI:** 10.1371/journal.pone.0001994

**Published:** 2008-04-23

**Authors:** Boris Zybailov, Heidi Rutschow, Giulia Friso, Andrea Rudella, Olof Emanuelsson, Qi Sun, Klaas J. van Wijk

**Affiliations:** 1 Department of Plant Biology, Cornell University, Ithaca, New York, United States of America; 2 Stockholm Bioinformatics Center, AlbaNova, Stockholm University, Stockholm, Sweden; 3 Computation Biology Service Unit, Cornell Theory Center, Cornell University, Ithaca, New York, United States of America; University of Oldenburg, Germany

## Abstract

Characterization of the chloroplast proteome is needed to understand the essential contribution of the chloroplast to plant growth and development. Here we present a large scale analysis by nanoLC-Q-TOF and nanoLC-LTQ-Orbitrap mass spectrometry (MS) of ten independent chloroplast preparations from *Arabidopsis thaliana* which unambiguously identified 1325 proteins. Novel proteins include various kinases and putative nucleotide binding proteins. Based on repeated and independent MS based protein identifications requiring multiple matched peptide sequences, as well as literature, 916 nuclear-encoded proteins were assigned with high confidence to the plastid, of which 86% had a predicted chloroplast transit peptide (cTP). The protein abundance of soluble stromal proteins was calculated from normalized spectral counts from LTQ-Obitrap analysis and was found to cover four orders of magnitude. Comparison to gel-based quantification demonstrates that ‘spectral counting’ can provide large scale protein quantification for *Arabidopsis*. This quantitative information was used to determine possible biases for protein targeting prediction by TargetP and also to understand the significance of protein contaminants. The abundance data for 550 stromal proteins was used to understand abundance of metabolic pathways and chloroplast processes. We highlight the abundance of 48 stromal proteins involved in post-translational proteome homeostasis (including aminopeptidases, proteases, deformylases, chaperones, protein sorting components) and discuss the biological implications. N-terminal modifications were identified for a subset of nuclear- and chloroplast-encoded proteins and a novel N-terminal acetylation motif was discovered. Analysis of cTPs and their cleavage sites of *Arabidopsis* chloroplast proteins, as well as their predicted rice homologues, identified new species-dependent features, which will facilitate improved subcellular localization prediction. No evidence was found for suggested targeting via the secretory system. This study provides the most comprehensive chloroplast proteome analysis to date and an expanded Plant Proteome Database (PPDB) in which all MS data are projected on identified gene models.

## Introduction

Chloroplasts are essential organelles of prokaryotic origin and carry out a wide range of metabolic functions. The chloroplast genome only encodes for about 100 proteins, whereas the vast majority of the chloroplast proteome is encoded by the nuclear genome. These proteins are generally synthesized as precursor proteins with cleavable N-terminal chloroplast transit peptides (cTPs) [1]. Several subcellular localization programs, such as TargetP [2] are available that predict these cTPs, with the number of predicted chloroplast (plastid) proteins ranging from about 1500 to 4500 proteins [3], [4]. However, several known plastid proteins appear to have no obvious cTP, and chloroplast outer envelope proteins never have a cleavable cTP (for discussion see [5]–[7]. It was recently suggested that an *Arabidopsis thaliana* (from here on referred to as *Arabidopsis*) chloroplast protein (a carbonic anhydrase) takes an alternative route through the secretory pathway, and becomes N-glycosylated before entering the chloroplast [8]. It is possible that more chloroplast proteins follow this route. Large scale experimental plastid proteomics studies are needed to evaluate unusual targeting pathways and to provide new training sets to improve subcellular localization prediction.

Driven by developments in mass spectrometry (MS), the *Arabidopsis* chloroplast proteome has been analyzed by MS in combination with various protein fractionation techniques to assign proteins to chloroplast compartments (reviewed in [9]–[11]). Collectively, these studies identified 1090 proteins (counting 1 gene model per protein), with an overall cTP prediction rate of 60% by TargetP (data not shown). However, from manual evaluation we estimate that 300–350 proteins likely represent false positive identifications and/or non-chloroplast contaminations. This shows that uncurated experimental proteomics data from isolated subcellular compartments and localization predictors do not provide sufficient quality for localization. However, the combination of multiple independent proteomics experiments, ideally from all compartments of a cell, as well as cross-correlation to detailed functional and localization (eg. with GFP fusion proteins) studies may allow high quality subcellular localization and functional annotation [12]. Currently, this curation process cannot be fully automated and requires manual supervision. Thus more experimental work and curation is needed to obtain a more in-depth and accurate overview of the chloroplast proteome in *Arabidopsis.*

Protein accumulation levels within a cell, or subcellular compartment such as the chloroplast, span five to ten orders of magnitude. To understand chloroplast function and homeostasis and to accommodate systems biology approaches to model genetic and metabolic networks [13], it is important to determine protein accumulation levels. A recent analysis of the *Arabidopsis* stromal proteome used gel based quantification to rank the abundance of 240 stromal proteins spanning several orders of magnitude [14]. The challenge is now to obtain accurate quantification for a larger percentage of the chloroplast proteome. Recently, large scale MS-based studies for yeast, humans, *E. coli* and other sequenced organisms have shown that the number of MS/MS spectra matched to a protein (spectral counts - SPC) positively correlates with the protein abundance [15]–[18]. Upon control of several experimental conditions, careful and stringent spectral assignments, and sophisticated normalization procedures, it appears that MS-based quantification can provide an attractive and sensitive tool to obtain large scale measurements of relative protein concentrations. For further review and discussions were refer to [19]–[21]. These new developments provide an excellent opportunity for quantification of the chloroplast proteome as will be demonstrated in the current study.

The half-life and function of proteins is often influenced by post-translational modifications (PTMs). N-terminal modifications of chloroplast proteins have shown to be important for chloroplast viability. For instance, N-terminal acetylation in the cytosol of nuclear-encoded chloroplast proteins is required for chloroplast function [22]. Furthermore, both chloroplast localized deformylase [23]–[26] and methionine endopeptidase are essential for *Arabidopsis* seedling viability [27], [28]. It is quite likely that these N-terminal modifications improve protein stability [29], for example to avoid degradation by the abundant chloroplast Clp protease system [30]. However, no systematic experimental analysis of N-termini of *Arabidopsis* chloroplast proteins has been carried out so far. PTMs, such as N-terminal acetylation, typically lead to a well-defined change in molecular mass that can often be detected by high quality MS. The rapid improvements in MS instrumentation, exemplified by the linear ion trap triple quadropole (LTQ) Fourier transform ion cyclotron resonance and LTQ-Orbitrap instruments, now facilitate a high throughput PTM analysis [31]–[35].

The current study determines chloroplast stromal protein abundance and N-terminal modifications, re-evaluates chloroplast transit peptides and cleavage sites, and provides a comprehensive catalogue and annotation of the chloroplast proteome, encompassing existing literature. The plastid proteomics database, PPDB (http://ppdb.tc.cornell.edu/), first described in [36], is focused on the (cell-type specific) chloroplast proteomes from maize and *Arabidopsis* and their functional annotation. We recently renamed the Plastid PDB into Plant PDB to better reflect the content. The dataset obtained in the current study is integrated in the PPDB, is expected to serve the plant community in small and large scale analyses where protein subcellular location, protein modification, function and abundance are important. Moreover, based on our experimental and theoretical analysis of the N-terminal portions and cTP cleavage sites, it is expected that the chloroplast data set presented here will facilitate improvement of subcellular protein localization predictors. Finally, the protein coverage and abundance of key chloroplast pathways and processes is discussed. This study demonstrates that ‘spectral counting’ can provide large scale protein quantification for *Arabidopsis*, which is important in the context of plant systems biology [13], [37].

## Results

### Experimental identification of the chloroplast proteome by LTQ-Orbitrap

To identify stromal and thylakoid proteins in the chloroplast, three independent *Arabidopsis* chloroplast preparations from mature rosette plants were used. Each preparation was separated into a soluble stromal and membrane fraction and then resolved by 1D SDS-PAGE, followed by in-gel trypsin digestion and identification by online nano-liquid chromatography tandem mass spectrometry (nanoLC-MS/MS) with an LTQ-Orbitrap, using data dependent acquisition (DDA) and dynamic exclusion. Biological replicates were analyzed two or three times to determine the technical variation of MS acquisition, as discussed further below.

Filtering criteria for MS-based protein identification were chosen to give an overall peptide false discovery rate (FDR) of 1%, as estimated by concatenated target/decoy database search [38]. Furthermore, only one gene model per protein was selected, which was either the one with the highest protein identification Mowse score, or in case of equal scores, the one with the lowest model number (indicated by the number after the digit). If the same set of peptides could be used to identify several different proteins, these homologous proteins were reported as an ambiguous group with all possible proteins listed, yet counted as one protein identification. For example, AT5G38410.1 and AT5G38420.1, corresponding to Rubisco small subunits 3B and 2B, were counted as one identification because they were described by the same set of peptides. Proteins were not reported if they could only be identified based on subsets of peptides matching to another identified protein. A more detailed description of the filtering procedures is provided in the Materials and Methods section.

**Figure 1:**
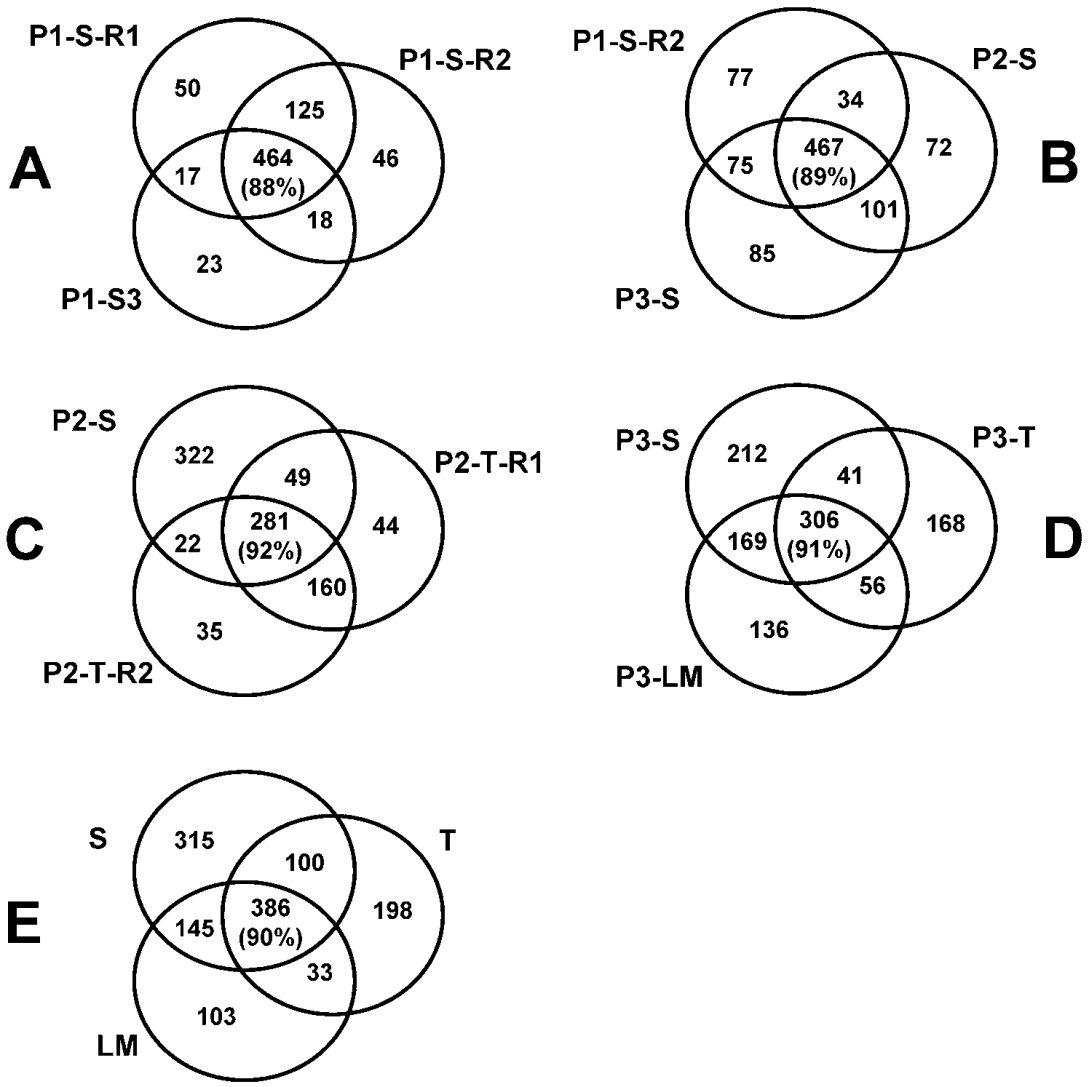
Identification of Chloroplast Proteins by nanoLC-LTQ-Orbitrap MS/MS. Venn diagrams show overlap between proteins identified in different preparations. Letter S denote soluble fractions, letter T denotes membrane fractions, letters LM denote low density membrane fraction, (A) Proteins identified in three technical stromal replicates of Chloroplast preparation 1, (B) Proteins identified in three biological replicates of stromal samples – chloroplast preparation 1 (technical replicate 2), chloroplast preparation 2, and chloroplast preparation 3, (C) Proteins identified in stromal and membrane (two technical replicates thereof) fractions of Chloroplast preparation 2, (D) Proteins identified in the three different fractions of Chloroplast Preparation 3, (E) Overlap between three sample types from all replicates combined. Number in parentheses is the percentage of proteins localized in chloroplast according to TargetP.

Overall, 1258 proteins were identified unambiguously. In addition, 22 pairs or small groups of homologous proteins were identified (Table S1). Table 1 and Figures 1A–E summarize the identification results and show overlap between the experiments. The number of identified proteins from individual mass-spectrometry analyses were as follows: i) The stromal fraction from chloroplast preparation 1 (P1) was analyzed three times, yielding 656, 653, and 522 proteins each, ii) stromal fractions from P2 and P3 were analyzed once each, yielding 674 and 728 proteins, respectively; iii) fractions enriched in thylakoid membrane proteins were analyzed twice for P2 (534 and 498 identifications), and once for P3 (571 identifications). Additionally, a low density membrane fraction obtained by high speed centrifugation of stromal extract was analyzed for P3, yielding 667 identifications (Table 1).

**Table 1:** Identification of Chloroplast Proteins by nanoLC-LTQ-Orbitrap.

Preparation^a^	IDs^b^	%TargetP^c^	Total Peptides^d^	Total Spectrum Counts^e^	Total Unique Spectrum Counts^f^	Combined Mowse Score
		C|S|M|O				
Prep1/S/R1	656	84.9|5.0|1.1|9.0	4464	8666	7289	253125
Prep1/S/R2	653	85.3|4.4|1.1|9.2	4488	8829	7433	256159
Prep1/S/R3	522	86.0|4.4|1.1|8.4	3392	6363	5433	173143
Prep2/S/R1	674	83.0|6.4|2.0|8.6	5212	12047	9995	417978
Prep2/T/R1	534	85.0|5.6|1.5|7.9	3414	8718	7506	289782
Prep2/T/R2	498	85.5|5.0|1.4|8.0	2977	7486	6427	268834
Prep3/S/R1	728	82.8|5.4|1.4|10.4	8006	18130	15373	707427
Prep3/T/R1	571	86.9|4.2|1.2|7.7	4227	9413	7637	360831
Prep3/LM/R1	667	77.1|6.4|3.0|13.5	3051	5874	5230	239411
Combined	1280	74.1|7.3|3.2|15.4	39231 (9262)	85526	71022	2.967*10^6^

a Three independent chloroplast preparations (∼30 plants used for each); S – enriched for chloroplast soluble proteins; T-enriched for thylakoid proteins; LM – low-density membrane fraction; R – denotes technical replicate.

b Number of un-ambiguous protein identification, *i.e.* several proteins that have the same set of peptides, are counted as one identification; only one gene model per accession was selected.

c Percent of proteins predicted by TargetP; Chloroplast (C), Signal peptide (S), Mitochondria (M), Other (O).

d Number of identified unique peptide sequences (ignoring charge state); number of unique peptide sequences (ignoring different charge states) is shown in parentheses for the combined dataset.

e Number of tandem MS spectra matched to peptide sequences.

f Number of tandem MS spectra associated with unique peptides; *i.e.* those peptides that are not shared between different accessions. Peptides representing a protein ambiguous group, which was reported as one identification were considered unique for this group.

To further assess the reproducibility between technical and biological replicates, we then carried out a G-test of independence of unique number of spectral counts (SPC) for each protein [39] [17], [40]. This shows that the technical variation resulting from on-line chromatography and MS analysis is very small, while the biological variation was mostly due to undersampling of low-abundant chloroplast proteins and infrequent observation of non-chloroplast contaminants (see Text S1 for details).

### Abundance of stromal proteins

It has been shown in DDA LC-MS-based analyses of digested protein mixtures that the number of MS/MS of spectral counts (SPC) correlates with protein abundance [15]–[18]. The matched spectral counts for each protein need to be normalized for protein properties to obtain an accurate abundance measurement. The most promising methods of normalization have been protein length [41] or number of theoretical and relevant tryptic peptides, possibly corrected for their propensity to be observed [18], [42].

To estimate abundance levels of the 946 proteins identified in the three stromal preparations, we calculated first the abundance factor (AF) from unique counts, and subsequently normalized AF for number of observable tryptic peptides yielding NAF. In cases where two or more homologous proteins were identified with shared and unique peptides, the number of spectra from shared peptides assigned to each protein was determined based on the ratios of spectra derived from the unique peptides that identified each protein, according to [43] (see Material and Methods for details). Shared counts contributed only once to the overall normalization.

**Figure 2:**
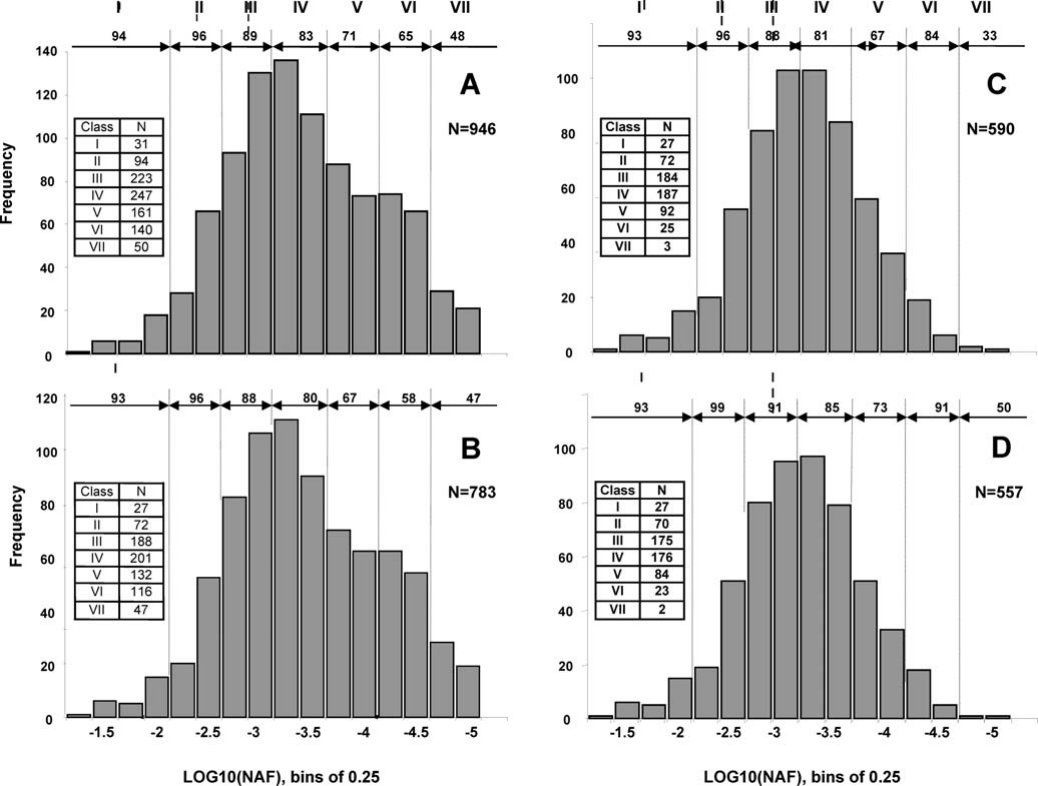
Frequency distribution of relative concentrations of soluble chloroplast proteins. Log10 abundance of stromal proteins were calculated from normalized SPC and corrected for predicted full tryptic peptides of the mature proteins within mass window of 700–3500 Da. Corrections were made for shared peptides as described in the Material and Method section. Each bin on the x-axis corresponds to 0.25 order of magnitude, with the total population spanning five orders of magnitude. The bins are grouped into seven abundance classes (I–VII, with I representing proteins of highest abundance). The percentage of chloroplast predicted nuclear-encoded proteins by TargetP (% cTP) is indicated. (A) Frequency distribution for the 946 proteins in the initial (unfiltered) dataset from stromal samples, (B,C,D) Frequency distribution of stromal protein after application of successive filters, as follows i) after known non-stromal chloroplast proteins (*i.e.* thylakoid, lumen, envelope) were removed (B), when only including proteins observed in 2 or more independent preparations (C), after removal of non-chloroplast contaminants (D).

Figure 2A shows the frequency distribution of the Log_10_ values of the NAF corrected for number of tryptic peptides for the 946 proteins in the initial unfiltered dataset from stromal samples. Each bin on the x-axis corresponds to a 0.25 order of magnitude, with the total population spanning five orders of magnitude. The bins are grouped into seven abundance classes (I–VII, with bin I representing proteins of highest abundance). The percentage of chloroplast predicted nuclear-encoded proteins is indicated, with 80% predicted cTP for the complete set. The decrease in cTP% coincides with the decrease in abundance, which is expected since most of the non-chloroplast contaminants (they have no true cTP) should be present at the lower end of the abundance spectrum.

With the objective to get a more accurate quantification of the actual stromal proteome, we applied a series of filters to the initial data set (Figures 2B–D). Figure 2B shows the frequency distribution of the protein abundance after known non-stromal chloroplast proteins (*i.e.* thylakoid, lumen and envelope) were removed. Furthermore, when we only consider proteins observed in 2 or more independent preparations, 193 proteins are removed mostly from abundance classes IV-V-VI-VII (Figure 2C). Cross-referencing these 590 remaining proteins to the existing literature suggested that 33 of these proteins were non-chloroplast contaminants (Table S2). These were subsequently removed to yield the final set of 557 proteins. The percentage of predicted cTP for these remaining proteins then increased to 87% (Figure 2D and Table S2). The validity of the filtering is supported by the observation that the abundance distribution becomes closer to normal (compare Figure 2A with Figure 2D), in agreement with the central limit theorem [44].

**Figure 3:**
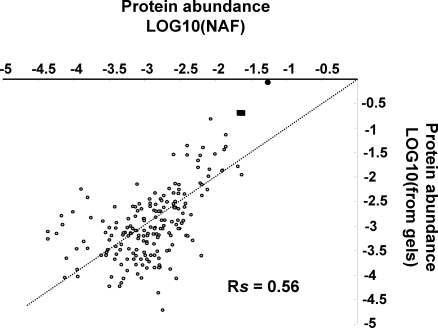
Cross-correlation between relative concentrations of stromal proteins quantified by spectral counting and by image analysis of gel separated proteins. Stromal protein quantified in a previous study from image analysis of stained 2-dimensional native gels (weighted for experimental protein mass) [14] were directly correlated to MS based quantified from the current LTQ data set. Log10 abundance of stromal proteins were calculated from normalized SPC and corrected for predicted full tryptic peptides of the mature proteins within mass window of 700–3500 Da. This showed strong positive correlation as indicated by a Spearman correlation coefficient of 0.56.

In a previous study, we quantified accumulation levels of 214 chloroplast stromal proteins (88% cTP) from image analysis of stained 2D native gels (weighted for experimental protein mass), covering ∼4 orders of abundance [14]. To evaluate how the current MS based quantification compares to this gel-based quantification, we cross-correlated the abundance of stromal proteins accurately quantified in both studies (Figure 3). This showed a strong positive correlation, as indicated by a Spearman correlation coefficient of 0.56. It appears that the spectral counting technique underestimates the most abundant proteins, in particular for the Rubisco large and small subunits. However, the gel based method is likely less accurate for the lower abundance proteins, as the signal/noise ratio for spot intensity was low – see [14]. When using protein length rather than observable tryptic peptides, the Spearman correlation was slightly lower (0.53) (not shown).

#### Coverage and abundance of chloroplast stromal functions and pathways

**Figure 4:**
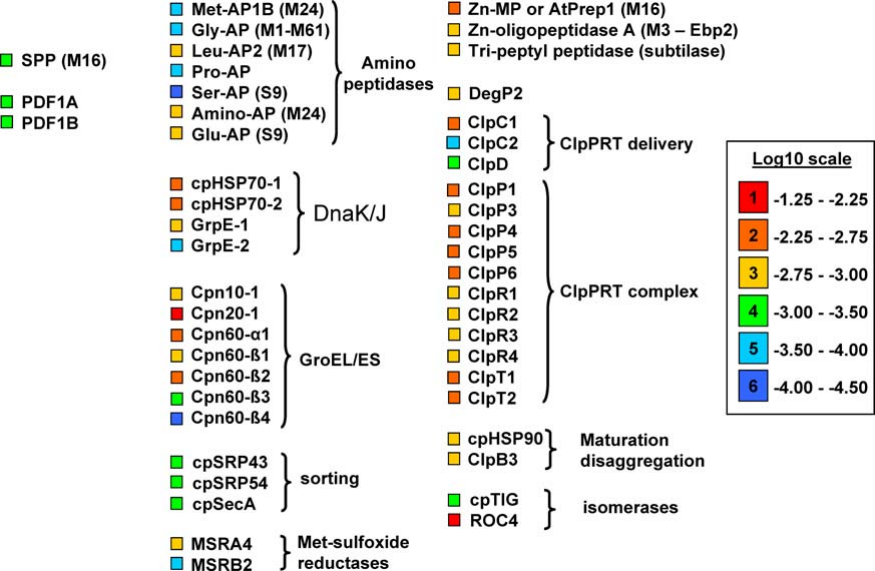
Quantification of the chloroplast protein homeostasis network including processing, (un)folding, maturation and proteolysis. Relative concentrations of 48 stromal proteins involved in the post-translational protein homeostasis network are displayed with color coding. Abbreviations are as follows: SPP, general stromal processing peptidase; PDF1A,B, methionine deformylases 1A,B; MSRA4,B2, methionine sulfoxide reductases; AP, amino-peptidases; CPN10,20,60, chaperone 10,20 and 60 of the GroEL/ES system; cpHSP70, heat shock protein 70; GrpE, nucleotide exchange factor; HSP90, heat shock protein 90; ClpB3, chaperone B3; cpSRP – chloroplast signal recognition particle subunit 43 and 54, involved in protein targeting components cpSRP43, cpSRP54; cpSecA – ATP-dependent Sec targeting component; cpTIG, a homologue of *E. coli* trigger factor involved in protein folding at the ribosome; ROC4, protein isomerase with unknown function; ClpP/R/S,T,C,D- subunits of the complete Clp protease system, DegP2 – protease of the Deg family; AtPrep1 - a Zn-protease suggested to be involved in degradation of processed cTPs; Zn-oligopeptidase A, homologue of a peptidase that in *E. coli* was suggested to degrade small peptides down-stream of the Clp protease system; TPPII, tripeptyl peptidase.

To obtain better insight in the role of the quantified stromal proteome, all proteins were (re)evaluated for function, using information from papers, functional protein domain predictions, and other resources (*e.g*.TAIR). We used the MapMan bin system [45] for functional classification. As compared to previous chloroplast proteome studies, the current study significantly increased coverage of lower abundant pathways. Examples are nucleotide synthesis and degradation and nucleotide transfer, represented by 22 proteins out of the 39 cTP predicted plastid proteins assigned to this pathway, with an average abundance (log10(NAF)), of −3.4. Also, a set of 14 low abundant t-RNA synthetases were observed in the stroma with an average of −3.6, while soluble proteins involved in tetrapyrole biosynthesis have an average abundance of −3.3. The quantified stromal proteome also has a high number of proteins involved in protein translation, (un)folding, targeting, processing, aa modifications, and proteolysis. Here we highlight those stromal enzymes involved in the post-translational protein homeostasis network steps, including N-terminal processing and modifications (Figure 4).

We quantified 48 proteins involved in the post-translational protein homeostasis network steps. They include the general stromal processing peptidase (SPP), both known methionine deformylases (PDF1A,B), two methionine sulfoxide reductases, seven amino-peptidases, the GroEL/ES and DnaK/J chaperone systems, HSP90 and ClpB3, protein targeting components cpSRP43, cpSRP54 and cpSecA, a homologue of trigger factor (involved in protein folding at the ribosome), the very abundant isomerase (ROC4), the complete Clp protease system (with the exception of ClpS), DegP2 protease, AtPrep1 protease suggested to be involved in degradation of processed cTPs [46], an oligopeptidase A, - a homologue of a peptidase that in *E. coli* was suggested to degrade small peptides down-stream of the Clp protease system [47]- and finally, a tripeptyl peptidase, TPPII (Figure 4). TPPII was shown to exist as a soluble, approximately 5- to 9-MDa complex and it was suggested that it is an exopeptidase that assists in aa recycling [48]. These proteins span 3 orders of magnitude, with Roc4 and Cpn20 being the most abundant and some of the amino-peptidases being the least abundant. The average coefficient of variation for abundance for this group of 49 proteins was 56%, which is excellent given that the abundance range of this protein population spans 3 orders of magnitude. These data are consistent with previous quantification of the ClpPRS system ([49], [50] and HSP70 and Cpn60 systems [14]. Considering the central role of SPP in processing of all incoming nuclear–encoded chloroplast proteins, it was interesting to note that its abundance is relatively low as compared to the chaperone systems. This suggests that the contact period between SPP and its substrates is relative short compared to the chaperone-substrate interactions.

### Chloroplast stromal proteome analysis by Q-TOF

In addition to the experimental proteomics data using the LTQ-Orbitrap, we also analyzed seven unpublished independent stromal preparations using a Quadropole-Time-of-Flight instrument (Q-TOF), a high quality, but older generation instrument with lower sensitivity and speed than the LTQ-Orbitrap. These seven independent experiments identified a total of 623 non-redundant proteins (1 gene model) with 1 or more unique peptides; 81% of these proteins had a predicted cTPs (Table S3). The false positive protein identification rate for proteins identified with 2 or more unique peptides was zero. When also including groups of proteins identified with only shared peptides (but not with subsets of peptides identifying other proteins), the total number of ‘identified’ proteins is 720 with a 77% cTP prediction rate (not shown). These data were used only for the evaluation for subcellular protein location, but not used for quantification, nor for PTM analysis, as will be discussed further below.

### Assigning proteins to the chloroplast based on spectral data?

Using the protein datasets from the ten biological replicates analyzed by LTQ-Orbitrap or Q-TOF, we tested if proteins could be assigned to the chloroplast based on the MS data alone. For that purpose, we merged each of the LTQ thylakoid and stromal data derived from the same chloroplast preparation. We then filtered the identified accessions in these LTQ and Q-TOF data sets each separately, using two simple and objective parameters: i) presence in at least 2 independent preparations, with at least 2 unique matched MS/MS spectra per preparation; the underlying assumption is that contaminations are often different in each independent preparation, and ii) at least 2 different peptide aa sequences across the independent observations. This is to avoid proteins that are identified with repeated matched MS/MS spectra (possibly in different charge states) of only 1 peptide sequence. Examples are AT5G39560 (WAVE protein) and At5G01730 (KELCH protein) each multiple times identified with peptides IEWINSVLTVPL and EDLISPR, respectively. Applying these two simple filters resulted in 739 nuclear-encoded proteins with an 86% cTP prediction rate and 46 chloroplast-encoded proteins in the LTQ-Orbitrap data sets and 340 nuclear-encoded proteins with a 91% cTP prediction rate and 13 chloroplast-encoded proteins in the Q-TOF stromal data sets (Table S4). Reassuringly, 96% of the proteins identified in this filtered Q-TOF dataset are also found in the filtered LTQ datasets, indicating that the chloroplast preparations were highly reproducible and consistent with the higher sensitivity of the LTQ-Orbitrap. Finally, we reanalyzed ‘in-house’ MS/MS data (from Q-TOF) that were underlying published analyses of thylakoid-associated lipoprotein particles or plastoglobules (11 biological preparations) [51] and of ‘stripped’ thylakoids (3 biological preparations) [52] and applied the same two filters for the data sets from each of the two types of preparations. Collectively, these highly filtered Q-TOF and LTQ datasets identified 762 nuclear-encoded (85% cTP) and 47 chloroplast-encoded non-redundant proteins (Table S4).

To evaluate our filter procedure, we determined potential non-chloroplast contaminants in this filtered dataset using an extensive cross-correlation to more than fifty published proteomics papers on *Arabidopsis* subcellular fractions, as well as information extracted from TAIR, from SUBA [53], and other literature. Details for this manual curation are explained in the Method section. Based on these cross-correlations, we found partially conflicting evidence for 21 proteins and we did not assign them to any subcellular location (Table S4). 30 proteins were considered non-plastid contaminations (Table S4). Therefore, 716 nuclear-encoded proteins (including 21 proteins dually targeted to plastid and mitochondria) were assigned to the chloroplast of which 89.5% have a TargetP predicted cTP. This dataset excludes groups of closely related homologues that we could not strictly distinguish by MS data alone.

### Extracting chloroplast/plastid proteins from the literature

In addition to the experimentally identified proteins, we also extensively screened the literature for likely plastid proteins. If the data provided for plastid localization were compelling (conclusive Western blot analysis or conclusive GFP/YFP localization), these accessions were also assigned to plastids. We did observe a significant percentage of these proteins by mass spectrometry, but sometimes they did not pass the requirement for multiple independent observations with at least two different peptide sequences, as discussed in the previous paragraph. In addition, several sets of well known chloroplast protein homologues (*e.g.* some members of the LHCII family) were now also included. In total 200 additional nuclear-encoded proteins were assigned to the plastid based on the literature (Table S4), bringing the total plastid assigned dataset to 916 nuclear-encoded proteins (86% cTP).

### cTP cleavage sites and subsequent N-terminal modifications

Once nuclear-encoded proteins are inside the chloroplast, the N-terminal cTPs are cleaved by the general SPP. Following cTP cleavage, chloroplast proteins possibly undergo additional cleavage by the various amino-peptidases (as discussed above) and N-terminal residues can be acetylated in an enzyme-catalyzed reaction in which the α-amino group accepts the acetyl group from acetyl-CoA [54]–[57]. For understanding protein function and location, and also for various practical applications (*e.g.* overexpression), identification of the correct N-terminus is important. Unfortunately, the only available cTP cleavage site predictor, which is part of TargetP, is not very precise since it was developed in 1999 based on a small training set available at the time [58].

To better define the cTP cleavage site and evaluate possible subsequent N-terminal PTMs, all MS data were reanalyzed allowing semi-tryptic peptides and optional N-terminal acetylation, using a narrow ±3 ppm precursor ion tolerance window in the search. We point out that N-terminal acetylation can not occur during sample preparation and occurs only enzymatically [59] and can thus be taken as evidence for an authentic N-terminus.

**Figure 5:**
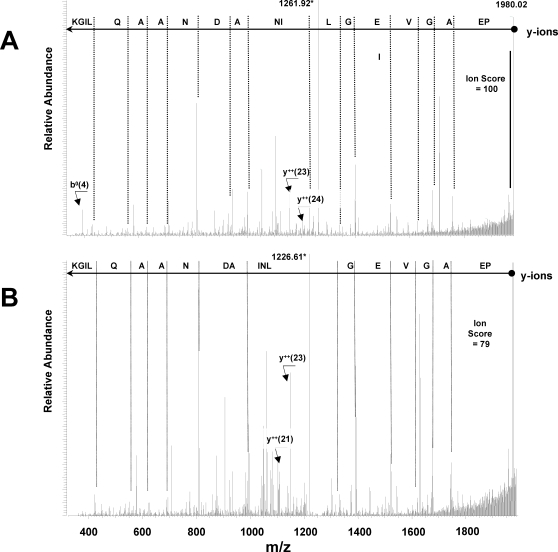
Tandem MS spectra of N-terminally acetylated peptides suggest presence of two isoforms of Cysteine Synthase, AT2G43750.1. (A) Tandem MS spectrum of doubly charged 25 aa-long, N-terminally acetylated AVSIKPEAGVEGLNIADNAAQLIGK peptide. Precursor ion is indicated with red asteric. Singly charged *y* ions are indicated by *blue* lines with corresponding aa residues shown on top - peptide sequence should be read *right-to-left*, starting with the most massive *y*(20) ion. Singly charged b ions are indicated by *red* lines with corresponding aa residues shown top – peptide sequence should be read *left-to-right*, starting with the lightest *b*(4) ion. Ions, whose presence strengthen the assignment of the N-terminal acetylation, *b^0^*(4), *y^++^(*23), and *y^++^*(24) are also indicated. (B) Tandem MS spectrum of doubly charged 24 aa-long, N-terminally acetylated VSIKPEAGVEGLNIADNAAQLIGK peptide. Precursor ion is indicated with red asteric. Singly charged *y* ions are indicated by *blue* lines with corresponding aa residues shown on top - peptide sequence should be read from *right-to-left*, starting with the most massive *y*(20) ion. Ions, whose presence strengthen the assignment of the N-terminal acetylation, *y^++^*(21), and *y^++^*(23) are also indicated.

We identified 47 N-terminal acetylated nuclear-encoded proteins (Table S5). Acetylation leads to an increase in retention time (∼6 min under our on-line LC conditions) and if both the non-acetylated and acetylated peptides are present, they can be identified as two peptides with different retention times and a mass difference of 43.018 Da. An interesting example is shown for cysteine synthase (Figure 5A, B). In this case, two acetylated N-termini were identified that differ by one aa in length. Figure 5A shows the MS/MS spectrum for the longer doubly-charged N-terminal peptide, Acetyl-AVSIKPEAGVEGLNIADNAAQLIGK, and Figure 5B shows MS/MS spectrum for the shorter doubly-charged N-terminal peptide, Acetyl-VSIKPEAGVEGLNIADNAAQLIGK. Both spectra are of high quality, with respective ion scores of 100 and 79, and with ions supporting assignment of the acetylation of the N-terminal residue (as opposed to lysines) present well above the noise level. The longer peptide was also observed in the triply-charged state in both non-acetylated and N-terminally acetylated forms eluting 6.4 minutes apart (spectra not shown). This example demonstrates that the assignments of acetylated residues are not false positives. It also demonstrates that either cTP cleavage by SPP can occur at more than one precise position, and/or that after the initial cTP cleavage by SPP, additional processing occurs by chloroplast amino peptidases, followed by N-terminal acetylation.

**Figure 6:**
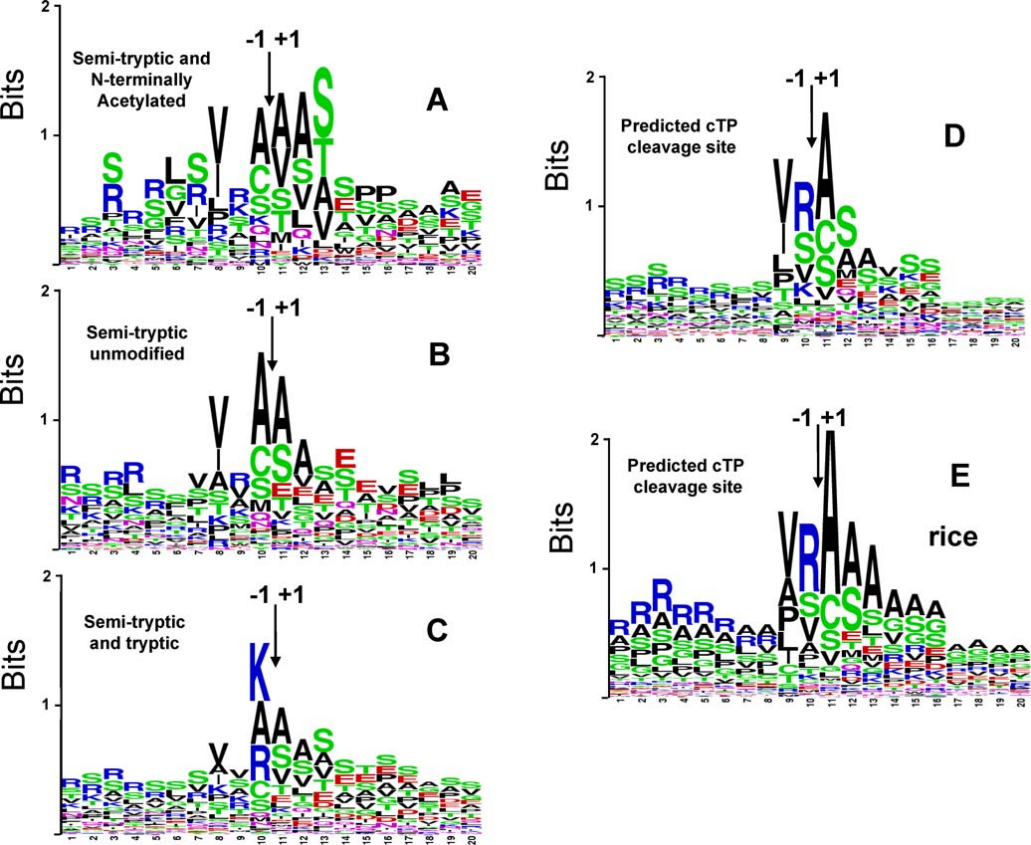
Consensus Sequences of the sites of cTP cleavage in chloroplast proteins. (A) Sequence logo of the cTP cleavage site, constructed for proteins, for which N-terminally Acetylated semi-tryptic peptides within 10 residues from predicted cTP cleavage site were observed by MS. N-terminally acetylated residue was assumed to represent true cTP cleavage site. (B) Sequence logo of cTP cleavage site, constructed for proteins, for which only non-modified semi-tryptic peptides within 10 residues from predicted cTP cleavage site were observed by MS. N-terminal residue of the semi-tryptic peptide closest to predicted cTP cleavage site was assumed to represent true cTP cleavage site. (C) Sequence logo of 203 stromal proteins for which the most N-terminal peptide (full tryptic or the semi-tryptic peptide) was within 10 residues of the predicted cTP, (D) Sequence logo of the predicted cTP of all 898 annotated *Arabidopsis* chloroplast proteins, but only those proteins (831) were used with a predicted cTP length of at least 20 aa residues. (E) Sequence logo of the predicted cTP of 802 rice proteins representing the best homologues for 898 annotated *Arabidopsis* chloroplast proteins, but only those proteins (714) were used with a predicted cTP length of at least 25 aa residues.

The protein sequences around their respective experimentally determined N-termini (±10 aa) from these N-acetylated proteins were aligned in a sequence logo plot (Figure 6A). This illustrates conservation at the −1 and −3 positions ([V/I] and [A,C,S] respectively), as well as the +1,+2 and +3 positions, with the +1 position representing the N-terminus. The +1,+2,+3 positions show a preference for [A/V/S]-[A/S/V/L]-[S/T/A/V] and likely represents a combination of a consensus motif for the SPP and the N-acetylase. Interestingly, while N-terminal acetylation is a very common N-terminal PTM in higher eukaryotes and lower eukaryotes, N-terminal acetylation is much less common in prokaryotes (bacteria and archaea) and relatively few acetylated N-termini have been determined [59]. Given the bacterial origin of the chloroplast and the prokaryotic-type chloroplast gene expression and protein homeostasis machinery, it is likely that acetylation patterns and the N-acetylases in chloroplasts are more similar to prokaryotes than eukaryotes. Most prokaryotic N-terminal acetylated proteins are ribosomal proteins, with A, M, S being most frequently acetylated, and P, T, G and V less frequently acetylated. The second residue has a strong affect on the frequency of acetylation, and in eukaryotes, acidic residues (D, E) stimulate acetylation, while P and basic residues (K and R) inhibit acetylation [59].

The prevalence of A,V,S,T (and M) as acetylated residues in our chloroplast proteins suggests that acetylation is carried out by a so-far unidentified NatA type acetylase. Interestingly, the second position for these sequences is occupied by A, S, V or L which is different from the preference in eukaryotes, although none of these residues have inhibitory effects on acetylation in eukaryotes. Additionally, the +3 position is occupied by the small, uncharged residues, S,A,V, and T, in agreement with avoiding charged and very large residues in eukayotes [59].

To distinguish between motifs for the SPP and the acetylase, we also assembled those proteins for which the most N-terminal identified peptide was within 10 aa residues from the predicted cTP cleavage site (upstream or down-stream) and for which the N-terminal residue was un-modified. The sequences of these proteins were aligned, assuming that the most N-terminal identified peptide represented the true N-terminus of the protein, resulting in a sequence logo plot of 62 proteins (Figure 6B). The upstream residues at the −3 and −1 positions are similar to the sequence logo plot from acetylated proteins (Figure 6A), but with stronger conservation of the A at the −1 position. The residues at the +1 and +2 position were enriched for A, S, but there was little conservation at the +3 position. This suggests that the conservation at the +3 position in Figure 6A was due to preference for the N-terminal acetylase, rather than SPP. This helps to define the consensus motif for N-acetylation in chloroplasts, which is important for future N-acetylation predictions. Interestingly, the set of acetylated proteins (Figure 6A), but not the unmodified set (Figure 6B), showed also some conservation at the −3 to −7 positions.

There is a potential pitfall in this analysis using tryptic digests (cleaving C-terminal of R and K), in that the −1 position is enriched for R or K. However, using semi-tryptic peptides, rather than full tryptic peptides suppressed this potential pitfall, which is obvious from the weak ‘conservation’ lack of R or K at the −1 position in the sequence logos. We note that the average length of the peptides in our analysis was ∼15 residues, explaining why there is little risk for artificial enrichment for R or K within the first 10 residues down-stream of the cleavage site. To illustrate this point, we created a sequence logo of 203 stromal proteins for which the most N-terminal peptide (full tryptic or the semi-tryptic peptide) was within 10 residues of the predicted cTP (Figure 6C); this clearly shows an ‘artificial’ enrichment of R and K at the −1 position, which has no biological relevance.

The sequence logo of the original experimental training set showed a consensus sequence for the cTP cleavage site of [V/I][R/A/V][A/MC]↓[A/S/M]. However, using the same proteins to predict the cTP consensus motif with ChloroP produced a consensus sequence of [V/I/P][R/S/V]↓[A/C/S][S/A/M], essentially moving the cleavage site one residue upstream [58]. When creating a sequence logo of the predicted cTP from a much larger dataset of 898 annotated *Arabidopsis* chloroplast proteins (outer envelope proteins were removed) (see further below), we observe a similar consensus sequence as for the original training set and a similar upstream shift of one residue as compared to the experimental consensus sequence presented. Moreover, the sequence logo of the predicted cTP of our large dataset shows a similar enrichment for R at the −1 position as originally observed (Figure 6D). However, our experimental data (Figure 6A,B) suggest that there is not such a strong preference for an R. The one residue upstream shift by the cTP cleavage site predictor, is most likely a consequence of the build-in preference (based a small set of *in vitro* cleavage experiments) for cleavage after an R, as explained in [58]. Based on our experimental data and the low abundance of the amino peptidases quantified in our study, it seems unlikely that all imported and SPP processed proteins undergo systematically a one (or two) aa removal from the N-terminus. We suggest that there is an excellent basis and larger experimental data set for improvement of the cTP cleavage site predictor.

### lTP cleavage site analysis

So far, 66 nuclear-encoded proteins assembled from various papers have been identified in the chloroplast lumen (http://ppdb.tc.cornell.edu/subproteome.aspx). These proteins have a lumenal transit peptide (lTP) down-stream of the cTP which is needed to cross the thylakoid membrane. In addition several integral thylakoid membrane proteins also have an lTP.

The LTQ-Orbitrap analysis identified 46 out of these 66 lumenal proteins, and except for the lumenal PSI subunit psaN (AT5G64040; see further below), none of them showed N-terminal acetylation, consistent with cleavage of the lTP on the lumenal side of the thylakoid membrane and the lack of a known acetylase in the lumen. This also confirms the low FDR for acetylation. In addition, we identified five integral thylakoid proteins with known lTP (Table 2). For 13 of these lTP proteins, we identified the processed N-terminus that exactly matched the predicted cleavage site and/or N-terminal Edman sequencing data from previous studies (Table 2). For one accession (AT3G01480 - TLP40), we found that the most N-terminal peptide matched the Edman sequencing data, but was 11 residues downstream of the predicted lTP. This either suggests an unusual lTP cleavage after the residues ‘DVS’, instead of after the predicted residues AHA, or this suggests that there was an additional cleavage event. Interestingly, the most N-terminal peptide for OEC-23-like (AT2G39470) and an isomerase (AT1G18170) were upstream of the predicted lTP cleavage site (Table 2), indicating either incorrect lTP cleavage site prediction or identification of an intermediate processing step. The N-terminal Edman sequence (ASA↓NAGVI) of psaN, coinciding with an lTP cleavage site, was 2 residues down-stream of the N-acetylated and unmodified peptides that we identified multiple times by MS. This suggests that biogenesis of psaN is more complicated that anticipated.

**Table 2:** Analysis of N-termini of lumenal proteins and potential new lumenal proteins.

Accession	curated protein name^a^	predicted start^b^	Edman^c^	Edman start^d^	start MS/MS^e^	predicted start to MS/MS start^f^	Most N-terminal Experimental MS/MS peptide
AT1G18170.1	Isomerases TAT lTP	86			61	−25	NVETTDWVASSLTR
AT2G39470.1	OEC23-like Tat lTP	107			87	−20	SYSPFVDR
AT4G24930.1	thylakoid lumen 17.9 kDa protein	64	IPSLS	64	64	0	IPSLSSSQPLTTPFTQSK
AT1G76100.1	PC-2	73	MEVLL	73	73	0	MEVLLGSDDGSLAFVPSEFTVAK
AT1G06680.1	psbP OEC23 Tat lTP	78	AYGEA	78	78	0	AYGEAANVFGKPK
AT5G23120.1	HCF136 Tat ltp	79	DEQLS	79	79	0	DEQLSEWER
AT1G20340.1	plastocyanin-1 (PC-1)	69	IEVLL	69	69	0	IEVLLGGGDGSLAFIPNDFSIAK
AT1G03600.1	PSII Lumen Tat lTP	69	AEDEE	69	69	0	AEDEEYIKDTSAVISK
AT4G02530.1	thylakoid lumen protein TL16.5	74	AILEA	74	74	0	AILEADDDEELLEK
AT3G56650.1	OEC23-like-1	68	REVEV	68	68	0	REVEVGSYLPLSPSDPSFVLFK
AT1G31330.1	psaF- subunit III	68			68	0	DISGLTPCK
AT1G50250.1	FtsH1	87			87	0	VVDEPASPSVVIESQAVKPSTPSPLFIQNEILK
AT1G54780.1	thylakoid lumen 18.3 kDa	85		85	85	0	SEFNILNDGPPK
AT5G42270.1	FtsH5 (VAR1)	77			77	0	VNEPVQPPAPTITAEAQSPNLSTFGQNVLMTAPNPQAQSSDLPDGTQWR
AT5G64040.1	psaN - TAT LTP	119/85	NAGVI	85	87*	2	GVIDEYLER
AT4G21280.1	psbQ OEC16 Tat ltp	78		76	82	4	VGPPPAPSGGLPGTDNSDQAR
AT3G50820.1	psbO OEC33-like	85	EGAPK	84	90	5	RLTYDEIQSK
AT5G66570.1	psbO OEC33	86	EGAPK	85	91	5	RLTYDEIQSK
AT4G15510.1	OEC23-like-3 Tat lTP	105	STPVF	104	111	6	EYIDTFDGYSFK
AT1G20810.1	Isomerases - lumen	72	RERRS	71	79	7	VIPLEEYSTGPEGLK
AT3G01480.1	Tlp-40	83	VLISG	93	93	10	VLISGPPIKDPEALLR
AT1G12250.1	thylakoid lumen PPR protein	91			101	10	GEFGIGSAAQYGSADLSK
AT3G10060.1	Isomerases TAT lTP	83			95	12	LPESDFTTLPNGLK
AT1G14150.1	PsbQ domain TAT lTP	66			79	13	YFMPGLSPEDAAAR
AT2G30950.1	FtsH2 (VAR2 and Pftf)	83	FGQSX	208	97	14	FLEYLDK
AT1G06430.1	FtsH8 TAT lTP	74			90	16	FLEYLDK
AT4G09010.1	Putative Asc-perox lumen	83	ADLIQ	82	100	17	ILLSTTIK
AT5G53490.1	peptapetide repeat TL17.4 (PPR)	78	ANQRL	77	95	17	AFVGNTIGQANGVYDKPLDLR
AT3G27925.1	DegP1	106	FVVST	103	124	18	LFQENTPSVVYITNLAVR
AT4G39710.1	Isomerases	74			94	20	SGLGFCDLDVGFGDEAPR
AT2G43560.1	FKBP isomerase	74	AGLPP	76	95	21	ELENVPMVTTESGLQYK
AT5G52970.1	thylakoid lumen 15 kDa protein	63	KVGVN	75	88	25	EFTSVIDVADFLSNGQEK
AT3G15520.1	peptidyl-prolyl isomerase TLP38	115	VLYSP	114	143	28	IIQASLEDISYLLR
AT3G55330.1	OEC23-like-4 Tat lTP TL25.6	75	AESKK	74	110	35	VYKDVIEPLESVSVNLVPTSK
AT1G76450.1	OEC23-like-2 Tat lTP	81	ETNAS	80	118	37	SITAFYPQETSTSNVSIAITGLGPDFTR
AT2G44920.1	pentapeptide repeat	82	FKGGG	81	128	46	LLGASFFDADLTGADLSEADLR
AT5G11450.1	OEC23-like-6 Tat lTP TP21.5	80			128	48	YSSAAPLSPDAR
AT5G45680.1	FKBP13 (involved with Rieske)	80	ETTSC	79	140	60	IGVGEVIK
AT4G18370.1	DegP5	74	XEQXX	71	152	78	LATDQFGLQR
AT5G39830.1	DegP8	91	LGDPS	90	220	129	VDAPETLLKPIK
AT1G08550.1	Violaxanthin Deepoxidase (VDE)	n/a	VDALK	113	254	n/a	TLDSGFFTR
AT1G77090.1	OEC23-like-5 Tat lTP TL29.8	n/a	VVKQG	71	82	n/a	VPGLSEPDEEGWR
AT3G60370.1	Isomerases	n/a	KTKSK	67	90	n/a	ENNAPDEFPNFVR
AT4G05180.1	psbQ OEC16-like Tat lTP	n/a	EAIPI	81	89	n/a	VGGPPLPSGGLPGTDNSDQAR
AT5G13410.1	Isomerases TAT lTP	n/a	SQFAD	89	89	n/a	SQFADMPALK
AT2G28605.1	PsbP domain - OEC23-like	n/a			99	n/a	AGANALFEELNNGSNNIGVVVSPVR
AT3G01440.1	PsbQ domain Tat lTP	n/a			126	n/a	NAFDLLAMEDLIGPDTLNYVK
AT3G24590.1	TPP-2 lumen	n/a			75	n/a	SAPSLDSGDGGGGDGGDDDKGEVEEK
AT4G28660.1	psbW -like	n/a			171	n/a	YSDQNGLQFVK
AT5G13120.1	peptidyl-prolyl isomerase TLP21	n/a			92	n/a	VYFDISVGNPVGK
AT1G05385.1	Psb27 cyanobacterial orthologue	68			150	82	
AT2G23670.1	expressed protein	72			72	0	
AT5G42765.1	expressed protein	65			86	21	
AT2G36145.1	expressed protein	63			75	12	

a curated protein name.

b predicted start (from cleaved cTP+lTP length).

c Edman sequence (from Peltier et al, 2002 or Schubert et al, 2002).

d Edman start position N-terminus by Edman.

e aa start position of most N-terminal MS/MS peptide. Bold numbers indicate perfect match to Edman sequence data and underlined numbers indicate perfect match to predicted lTP cleavage side.

f distance of most N-terminal MS/MS peptide to cleaved lTP site (negative is upstream of lTP cleavage site).

* peptide observed in acetylated and unmodified form.

### The search for new lumenal proteins

Genome-wide predictions [4], [60] and manual inspection [61] suggest that there are possibly another 20–100 unknown lumenal proteins. Therefore we investigated those identified proteins for which the most N-terminal (tryptic/semi-tryptic and/or modified) peptide matched the predicted lTP cleavage site or was within 100 residues downstream of the predicted lTP, without any additional peptides matching upstream of the predicted lTP site. These proteins were then evaluated for experimental mass spectrometry data and other information. This identified four putative lumenal proteins with unknown function, three of which were also identified in our earlier thylakoid analyses [36], [52] (Table 2). The predicted lTP cleavage site (SMA↓ENI) for one of the proteins (AT2G23670) coincides precisely with the most N-terminal peptide identified (ENIPLFGIR). Putative lumenal protein At1G05385 is an orthologue of cyanobacterial protein Psb27 which was suggested to be involved in assembly of the water splitting complex of PSII [62], [63], consistent with location on the lumenal site of the thylakoid. We note that Psb27 has another homologue in *Arabidopsis*, At1G03600, for which we previously determined the N-terminus by Edman sequencing and which we assigned to the lumen [60] and was recently showed to be involved in PSII assembly in *Arabidopsis* [64]. Putative lumenal protein AT2G36145.1 has no predicted function, nor predicted TMD and a maize homologue (TC294822) was found in maize thylakoids (Majeran et al, submitted). Putative lumenal protein AT5G42765 was identified with strong MS data in the thylakoid and the most N-terminal (semi-tryptic) peptide suggests an lTP cleavage site of AFA↓FSLGISGPK (Table 2).

### N-terminal modifications of chloroplast-encoded proteins

Chloroplast-encoded proteins are synthesized with an N-terminal formylated methionine residue. The N-termini of 59 chloroplast–encoded proteins from various plant species were experimentally determined by Edman sequencing across multiple studies, and subsequently assembled and evaluated [26] (see the table at http://www.isv.cnrs-gif.fr/tm/maturation/images/chloro.html). This showed that most N-formyl groups are removed by the chloroplast localized deformylases, PFD1B and possibly PFD1A [25], which were both quantified in our study (Figure 4). The deformylation is often followed by removal of the methionine, presumably by one or several of the chloroplast methionine aminopeptidases, MAP1B,C,D [23], [26]. In this study we only observed and quantified MAP1B (Figure 4), suggesting that MAP1B is more abundant than the other two amino peptidases.

Since no systematic experimental N-terminal analysis for chloroplast-encoded proteins in *Arabidopsis,* we used the LTQ analysis to evaluate information about the N-termini of 54 identified chloroplast-encoded proteins. For 12 chloroplast-encoded proteins we determined the N-terminus (Table 3), while the N-terminus for several others could not be determined, because they start with a short (semi-)tryptic peptide of less than 7 residues These short peptides are excluded from our database searches because of the relatively low information content. Four out of those 12 proteins (rpl14, and NDH subunits A,I,J) were identified with their N-terminal methionine. The N-termini of Rpl14 and NdhI were also observed with the methionine removed. In case of NDHA, the methionine was formylated; however, we must be careful as samples have been observed to become non-enzymatically formylated during the sample preparation [65]. For five proteins (cyt*b6*, rps8, rps15, D2, CF1ε), we observed that the methionine was always removed and two of those processed N-termini (for D2, CF1ε) were acetylated (both at a T residue). In case of CF1β and RBCL, the first two residues were removed and the new N-termini (respectively an R and P residue) were acetylated. Thus in addition to preferential N-acetylation of A,V,S,T,M observed for the imported nuclear-encoded proteins (Figure 6A), N-acetylation can also occur at T, R and P. Cyt*f* is the only c-encoded protein with a (SecA-dependent) lTP; this lTP has 36 residues. We did identify the processed N-terminus (starting at residue 37) of mature cyt*f* which was not acetylated, as expected since it is located on the lumenal side of the thylakoid membrane (Table 3). The MS based data were consistent with the data assembled or predicted in [26].

**Table 3:** N-terminal modifications of chloroplast-encoded proteins.

Accession	Protein name	Start MS/MS	most N-terminal sequence MS/MS	N-terminal Removal and Modification^a^
ATCG00780.1	50S rpl14 ribosomal protein	1	MIQPQTYLNVADNSGAR	formyl remove; variable Met (Ox)
ATCG01090.1	NDH I	1	MLPMITGFMNYGQQTLR	formyl removed; Met (Ox)*3
ATCG01100.1	NDH A (1)	1	MIIYATAVQTINSFVK	Met (Ox); possibly Formyl (N-term)
ATCG00420.1	NDH J	1	MQGTLSVWLAK	formyl removed; Met (Ox)
ATCG00720.1	petB - Cytochrome b6	2	SKVYDWFEER	formyl-met removed; none
ATCG00770.1	30S rps8 ribosomal protein	2	GKDTIADIITSIR	formyl-met removed; none
ATCG01120.1	30S rps15 ribosomal protein	2	IKNIVISFEEQKEESR	formyl-met removed; none, several modifications, N-term methyl.
ATCG00470.1	CF1e - atpE	2	acyl-TLNLCVLTPNR	formyl-met removed; N-terminal Acetylated
ATCG00270.1	psbD D2	2	acyl-TIALGKFTK	formyl-met removed; N-terminal Acetylated
ATCG00480.1	CF1b - atpB	3	acyl-RTNPTTSNPEVSIR	formyl-met removed; N-terminal Acetylated
ATCG00490.1	Rubisco large subunit	3	acyl-PQTETKASVGFK	formyl-met-S removed; N-terminal Acetylated
ATCG00540.1	cytochrome f (sP of 35 aa)	36	YPIFAQQNYENPR	lTP removed; none

Comments for each accession		Giglione et al 2001^b^
ATCG00780.1	Seen many times with good scores - methionine is not cleaved	formyl removed; MI (s)
ATCG01090.1	High ion score (83)	formyl removed; MI (t)
ATCG01100.1	High ion score (90)	formyl removed; MI (p)
ATCG00420.1	Seen many times with good scores - methionine is not cleaved	predicted formyl removed; MQ
ATCG00720.1	Methionine always cleaved -observed many times	formyl-met removed; S (s)
ATCG00770.1	observed only once	formyl-met removed; G (methylated) (s)
ATCG01120.1	Methionine is cleaved	formyl removed; MK (s)
ATCG00470.1	Methionine always cleaved -observed many times	formyl-met removed; S (c,w)
ATCG00270.1	Ion score 49; good MS/MS spectrum	T(phosphorylated)I (a,p,s)
ATCG00480.1	observed several times	formyl-met-removed; S(c)
ATCG00490.1	good scores	formyl-met-S removed;P (methylated) (me,s,t,w)
ATCG00540.1	semi-tryptic peptide	YP (position 36)

a N-terminal removal and subsequent modification of new N-terminal residue.

# From Giglione et al 2001. s-spinach; t - tobacco; p - pea; w- wheat a-Arabidopsis; c-cucumber.

### Are there chloroplast proteins that are targeted via the secretory system?

Recently there was a report that a chloroplast carbonic anhydrase (At3G52720) was targeted to the chloroplast via the secretory system [8]. It was shown that that this carbonic anhydrase was synthesized with an N-terminal sP and after cleavage in the ER, it reached the chloroplast through an unknown mechanism. It was hypothesized that this maybe a more common pathway for targeting of nuclear-encoded proteins to the chloroplast. Therefore, the 916 nuclear-encoded plastid proteins (but excluding 18 known outer envelope proteins) were passed through the SignalP predictor (http://www.cbs.dtu.dk/services/SignalP/). Only 0.6% of the proteins were predicted to have a sP, while negative test sets, *e.g.* cytosolic and mitochondrial proteins, showed similar or higher sP prediction rates (not shown). Therefore, it seems unlikely that a significant percentage of chloroplast proteins are targeted via the secretory system.

### cTP properties of chloroplast proteins and species specific differences

In addition to our current analysis discussed above, our previous analyses on smaller data sets [4], [14] also show that the true positive prediction rate for cTP prediction by TargetP (and other predictors) is ∼86%, despite various reports of lower true positive rates - for discussion see [2], [5]. Properties and aa composition of cTP have been extensively analyzed in the past [1], [58], but the ∼10 fold larger set of chloroplast proteins from this study provides an excellent opportunity to re-evaluate these properties.

**Figure 7:**
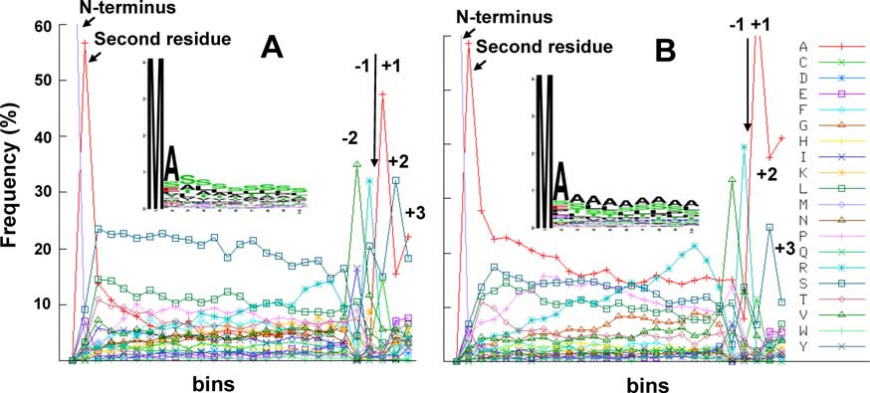
Chloroplast transit peptide analysis of *Arabidopsis* chloroplast proteins and predicted rice homologues. Sequence logos of the first 20 N-terminal residues (A,C) and aa distribution and frequency across the normalized (binned) cTP length (B,D) for 898 annotated *Arabidopsis* chloroplast proteins (A,B) and the 802 predicted rice homologues (C,D). Only those proteins were used with a predicted cTP length of at least 20 aa residues (831 and 714, respectively from *Arabidopsis* and rice).

We created sequence logos of the 20 most N-terminal residues (Figure 7A) and aa frequency distribution plots over the entire predicted cTP (Figure 7B) for the 916 annotated nuclear-encoded plastid proteins (excluding outer envelope proteins). A weak three domain structure is discernable: (i) an N-terminal domain beginning with MA, rich in S, L, A, and T, potentially terminating with P, (ii) a central domain lacking acidic residues (D/E) and with S, L, and P overrepresented but decreasing slightly towards the cleavage site, while R increases, and (iii) a C-terminal cleavage site domain enriched in R and with a loosely conserved motif VRA↓AA around the cleavage site. In contrast to previous observations [2], [66], the plots show that the boundary of the N-region is rich in P but not G, that acidic residues (D/E) are underrepresented across the entire cTP (except for the second position), and that apolar L (frequency is 10.4% in total *Arabidopsis* cTP set) and P (7.3%) are enriched, in addition to the hydroxylated residues S (19.3%) and T (6.9%). The plots also show that the second position is an A residue in only 57% of the cases (Figure 7A,B).

To investigate if there is a significant difference between *Arabidopsis* and rice chloroplast proteomes (two species about 100 million years apart in evolutionary distance), best rice homologues (E value<0.1) were obtained for the *Arabidopsis* set. This resulted in 802 rice proteins of which 74% had a predicted cTP (compared to 86% for the Arabidopsis proteins). Sequence logos and aa frequency distribution plots were generated similarly as for *Arabidopsis* (Figures 7C, D). This showed that the second position in plastid rice cTPs is enriched for A (about 59%), S, and E, followed by a clear preference for small uncharged residues A, S, and L (Figure 7C,D), a pattern very similar to *Arabidopsis*. On the other hand, a much higher abundance of A (18.0% in predicted rice cTP set versus 7.1% in predicted *Arabidopsis* cTP set), in particular, but also P, G, and R was observed in rice, while N, K, S, and T were less common in rice. Surprisingly, threonine is slightly underrepresented in these rice cTPs compared to its general abundance in proteins (Swiss-Prot release 54.0). Overall, this is in agreement with an earlier report for smaller datasets [57]. There is also a difference in the average length of the predicted cTPs: 50.4 residues for rice and 53.6 residues for *Arabidopsis,* p-value<0.0002 using Mann-Whitney test (excluding predicted cTPs shorter than 20 residues since these are likely wrong predictions). Moreover, the rice cTP content was slightly more conserved than for *Arabidopsis.* Finally, the predicted cTP cleavage site for the chloroplast rice homologues is very similar as for *Arabidopsis*, but with a stronger enrichment for A over S (Figure 6E). Our analysis suggests that specific rice and *Arabidopsis* cTP predictors might yield higher true positive rates and could also provide better predictions for other sequenced monocotyledons (*e.g.* maize, sorghum) and dicotyledons (*e.g.* poplar, medicago, tomato). Similarly, a specific cTP predictor for the sequenced green algae *Chlamydomonas reinhardtii* may help to alleviate the poor prediction success rates.

## Discussion

### The identified chloroplast proteome and its functions and criteria for contaminations

This study identified 1325 proteins in chloroplast samples, including ∼400 proteins not yet experimentally observed before. We used two MS platforms and 95% of the proteins identified in the stromal preparations by Q-TOF MS were also found in the LTQ-Orbitrap analysis. This clearly reflects the higher sensitivity and speed of the LTQ-Orbitrap platform, as was recently detailed [67], and also underscores the reproducibility of the stromal preparations.

Uncurated experimental proteomics data from isolated subcellular compartments do not provide sufficient quality for localization, as contamination of even less than 1% protein mass can result in significant numbers of identified proteins from other subcellular compartments. However, by demanding that proteins are identified with multiple high quality MS/MS spectra (with FPR <1%) and multiple unique peptide sequences in multiple independent preparations, many of these contaminations can be eliminated. We showed that this was particularly effective for chloroplasts. Subsequent comparison to proteomics studies of other subcellular compartments and cross-correlation with studies of localization and function removed (most) remaining contaminants.

Some 20 non-chloroplast proteins were frequently observed in our chloroplast preparations, with scores placing them in low abundance classes. For instance, we frequently observed peroxisomal proteins involved in photorespiration. Repeated analysis of total *Arabidopsis* seedling extracts by LTQ-Orbitrap MS as described here, show that these proteins are among the top scoring proteins in terms of matched MS/MS spectra (Zybailov, Friso and van Wijk, unpublished data). We also noted that most of these contaminants are observed in many proteomics studies of other organelles and membranes, further illustrating the benefits of cross-correlating different studies, made easy through an integrative PPDB. A recent mitochondrial proteome analysis observed many enzymes in the cytosolic glycolytic pathway and it was suggested that they interact tightly with mitochondria, explaining their frequent identification in mitochondrial samples [68]. A similar scenario may explain the identification of cytosolic HSP70 and an isomerase in the chloroplast preparations. This brings about the question how one can identify proteins that have less than five orders of magnitude abundance than the most abundant chloroplast proteins. We believe that it will be possible to assign such proteins to the chloroplast based on repeated MS based identification, when many other subcellular compartments are also analyzed with similar high sensitivity setups. A collective effort of the plant (proteomics) community specialized in preparing the various subcellular compartments will be needed to create a high quality ‘proteome map’ of the *Arabidopsis* cell.

The stringent filtering of our experimental proteomics data, combined with known chloroplast proteins from the literature, identified 916 nuclear-encoded chloroplast proteins. The experimentally identified proteins include many low abundant regulatory proteins, such as several kinases involved in aa biosynthesis (*e.g*. shikimate-kinases At2G35500 and At3G26900), a protein tyrosine phosphatase (AT3G44620) and the thylakoid integral membrane protein state-transition kinases, STN7 [69] and STN8 [70], not earlier observed in MS studies. In addition, we identified multiple low abundant putative RNA and DNA proteins and four RNA polymerases (Rpo-A,B,C1,C2) that were previously only identified by MS in experiments specifically focused on the transcriptional apparatus and the plastid chromosome (the nucleoid) [71] – here it is worth noting that we identified these polymerases in the thylakoid fraction, consistent with the thylakoid association of the plastid nucleoid. We also identified several new putative thylakoid lumen proteins that may help to clarify the function of this enigmatic compartment. The chloroplast protein dataset will be an important resource for detailed chloroplast studies, as well as large scale *Arabidopsis* analysis.

Ultimately, it would be ideal to identify every protein in the chloroplast, but given the anticipated wide range of protein abundance (>6 orders of magnitude) and the dynamics of the chloroplast proteome, this is not feasible. To better understand coverage and function of the identified chloroplast proteome, we compared the MapMan bin distribution (for functional assignments) of the cTP predicted plastid proteome (4053 - only 1 gene model) with the curated plastid proteome. This shows that the identified chloroplast proteome is overrepresented (as compared to the predicted plastid proteome) in co-factor and vitamin metabolism (59%), Photosynthesis (58%), N-metabolism (+54%), tetrapyrrole metabolism (47%), major CHO metabolism (+45%), redox regulation (+44%), oxidative pentose phosphate pathway (49%), while proteins involved in signaling (−74%), stress (−69%), development (−47%), unassigned (−45%) and RNA (−40%) are underrepresented. Even if functional assignments and cTP predictions are imperfect, this provides an impression of relative protein abundance of the various functional classes and processes within chloroplasts, with overrepresented and underrepresented proteins being respectively of higher and lower abundance. The actual quantification of the stromal proteome using the spectral counting technique provides a more accurate estimate of pathway abundance.

### Stromal proteome quantification and pathways analysis

Large scale quantification of protein abundance, alongside quantification of transcripts and metabolites, will be needed for systems analysis of *Arabidopsis* [13]. However, large scale protein quantification remains challenging, in particular due to the large expression range and proteome complexity [19]–[21]. In this study we show that ‘label-free’ MS based quantification does provide meaningful data and we provide a quantification protocol.

The abundance of 557 stromal proteins was determined using SPC that were either normalized for the length of each mature protein, or the number of theoretical tryptic peptides for each protein within the relevant mass range. The two normalization procedures resulted in comparable results, with the normalization by theoretical tryptic peptides yielding somewhat better accuracy, as determined from cross-correlation to published gel-based quantification of some 200 stromal proteins [14]. The cross-correlation showed that the ∼10–20 most abundant proteins were somewhat underestimated, as is expected when using DDA with dynamic exclusion (*i.e*. avoidance of repeatedly sequencing the same peptide). Quantification by spectral counting is likely to improve if a correction is introduced for the propensity of the theoretical peptides to be observed, as discussed in [43] [42]. Such propensity is dependent on the proteomics ‘pipeline’ (protein separation and MS platform) [42], [43]. The gel based quantification using native gels (not IEF) as the 1^st^ dimension and SDS-PAGE as the second dimension used in our previous study [14] is particularly good for quantification of the most abundant proteins. However, this gel based method fails to accurately detect and quantify the lower abundance proteins and also cannot accurately quantify proteins that co-migrate with other proteins. Gel based quantification is thus not a suitable alternative for large scale quantification. Additional (but far more costly and labor intensive) techniques, such as titration with known amounts of stable isotope labeled ‘proteotypic’ peptides uniquely representing each proteins will be needed to obtain precise quantifications (reviewed in [20]). Recently, quantitative MS using multiple reaction monitoring (MRM based quantification) without stable isotopes was demonstrated and may provide a good alternative for quantification of known sets of proteins [72]. It is clear that while further optimization of MS based quantification is beneficial, our current quantification of the stromal proteome using spectral counting does provide an excellent overview of over 550 stromal proteins, with relatively small variation and a dynamic range of four orders of magnitude. We used this quantitative data to highlight the post-translational protein homeostasis machinery, while the remainder of the dataset is provided as a community resource for functional analysis of the chloroplast.

### Analysis of the cTP and cleavage site suggests potential for improved subcellular localization prediction

Determination of the protein subcellular localization in *Arabidopsis* is important since it often dictates the function of a protein and it is also needed to calculate metabolic flux and contributions to signal transduction cascades (e.g. kinases, phosphates, etc) [73]. Accurate prediction of subcellular protein localization may ultimately alleviate the necessity to identify each protein experimentally. Currently, large scale experimental proteome studies are needed to provide larger training sets to improve such predictors and our study provides such a large, carefully curated, positive training set for the chloroplast.

Prediction of protein location in the chloroplast (plastid) is an important tool to identify proteins that so far escaped experimental identification, but true positive rates (∼86%) and false positive rates (∼35%) need to be further improved [2]. The large set of curated plastid proteins in this study and the detected features in the cTP cleavage site, suggest that there is a good basis for improving plastid localization prediction. Analysis of unfiltered sets of identified proteins in stromal samples suggested that the cTP success rate was much lower for low abundant proteins than for high abundant proteins. However, when chloroplast membrane associated and thylakoid lumen proteins, as well as non-chloroplast contaminants, were removed, this bias was strongly reduced, indicating that cTP prediction rates are relatively independent of protein abundance, although some bias for the very high abundant proteins (class I and II) does seem to exist, as also indicated by the 97% cTP prediction rate of those 156 proteins that were identified in each of the nine LTQ experiments (Table S6). We also observed clear indications that plastid proteins in rice and *Arabidopsis* are different in their cTPs, confirming less detailed and smaller scale analyses [57]. This warrants the development of species-specific (or dicotyledon and monocotyledon specific) plastid localization predictors. Retraining of the TargetP predictor based on information presented in this study, as well as new datasets from the recent literature is in progress (Emanuelsson, unpublished).

### N-terminal processing and modifications and their physiological role

The proteome complexity is increased by PTMs that play a role in protein location, protein-protein interactions and folding, enzymatic function and protein life-time. Taking advantage of the high accuracy of the Orbitrap, we were able to determine the N-termini of 47 acetylated nuclear-encoded chloroplast proteins and also identified processed (by deformylase and amino-peptidase activity) and unprocessed N-termini of chloroplast-encoded proteins. The biological significance of N-acetylation varies with the particular protein and has been shown to affect protein-protein interactions, protein assembly and enzymatic activities [59]. Analysis of the acetylated N-termini identified in the current study suggests that in case of nuclear-encoded proteins, small hydrophobic residues A, V, followed by small, hydroxylated residues S and T account for 80% of the acetylated residues. Position 2 is occupied by A, S, V, L and position 3 essentially by S,T,A,V. This illustrates that G, charged residues (D,E,K,R,H), aromatic residues (Y,N,Q) and residues with large side chains (I and L) are largely avoided in the first three positions. This includes primary destabilizing residues in *E. coli* (F,Y,W,L), as well secondary destabilizing residues (R,K) although the latter is likely a consequence of the tryptic digest. In case of chloroplast-encoded acetylated proteins, all but one were acetylated after f-Met removal. The acetylated N-termini residues are either T, R or P, with the secondary residues L,I,T,Q, which is very different from the residues in nuclear-encoded proteins. This could suggest involvement of two different N-acetylases, one operating co-translationally in case of chloroplast-encoded proteins and the other operating pos-translationally. We are not aware that anyone has investigated the role of acetylation of these or other chloroplast proteins, and this collection of acetylated proteins will provide a basis for such investigations.

The N-end rule states that the half-life of a protein is determined by the nature of its N-terminal residue. Whereas this fundamental principle is conserved from bacteria to mammals, prokaryotes and eukaryotes employ distinct proteolytic machineries for degradation of N-end rule substrates and the stabilizing and destabilizing residues are also different. The proteolytic machineries in plastids and chloroplast are all distinctly of prokaryotic origin, and it is therefore anticipated that the N-end rule stabilizing and destabilizing residues also follow bacterial rules. Proteolysis in prokaryotes and plastids is entirely independent of ubiquitin. Instead substrate selection is mediated by specialized HSP100 components (in chloroplasts ClpC1,C2, and ClpD) and aided with so-called adaptor proteins (ClpS), which together transfer the substrates to their cognate proteolytic partners. The N-rule and proteolysis in prokaryotes is best studied in *E. coli* [74], [75]. In *E. coli*, large hydrophobic (L.F,W,Y) and basic residues (R,K) represent primary and secondary destabilizing residues. Generally, M-aminopeptidases do not generate N-end rule destabilizing N-termini and indeed, the processed N-termini of the chloroplast-encoded N-termini followed this rule. In addition, in *E. coli*, proteins with an R or K as N-terminus can be destabilized by addition of L or F through an L/F-tRNA protein transferase – but no such transferase has yet been identified in chloroplasts. As mentioned above, we only observed one nuclear-encoded protein with an N-terminal destabilizing residue, Y in AT3G55250.1, which was observed multiple times but always carrying an N-terminal acetylation. Inspection of the sequence logos of the experimental, non-acetylated cTP cleavage site (Figure 6B) showed that the experimental N-termini essentially avoided destabilizing residues.

### Creating resources for the plant community

To disseminate our chloroplast proteomics data and integrate these with other types of proteomics information, we had developed the Plastid Proteomics Database, PPDB, initially reported in [36]. For this study we have added several features to the PPDB, in particular to better display details of MS based identification such that the user can better evaluate the significance of the protein identification and assigned localization. This includes mass error for all precursor ions and matched peptides, protein Mowse scores, as well as peptides with PTMs and a search option to extract all PTMs (with peptides and accessions). Importantly, all matched MS data are projected on the relevant gene models (using ‘pop-up’ windows), aiding in a better understanding of gene models and PTMs. MS data from earlier published papers (since summer 2004) were all re-searched and filtered and search data extracted and uploaded into PPDB. In addition, all protein accessions can be directly cross-referenced against identified proteins from more than 50 published proteomics papers (from *Arabidopsis* and other members of the *Brassicae* family), as well as the subcellular localization in GO (experimental) from TAIR, localization data from SUBA (http://www.suba.bcs.uwa.edu.au) and best matching homologues in maize and rice. Many of these data types can be directly exported from the PPDB as excel files. Since we also curated the location of hundreds of non-chloroplast proteins, and to reflect other available information, we changed the name from Plastid PDB to Plant PDB. Our PPDB is a unique resource for the plant community and complements other plant proteomics resources, such as Promex, a mass spectral reference database for proteins and protein phosphorylation sites [76], SUBA a database for *Arabidopsis* protein localization [12], as well as databases specialized in other organelles, such as peroxisomes [77]. We conclude that this study provides the most comprehensive chloroplast proteome analysis to date and a unique web-based *Arabidopsis* proteome resource.

## Materials and Methods

### Plant growth, chloroplast isolation and subfractionation

*A. thaliana* (Col 0) wt and *ffc1-2* [78] were grown on soil under short day (10/14 hours light/dark) with 280 µmol photons.m^−2^.s^−1^ light at 23/19°C in controlled growth chambers (Conviron). *clpr2-1* [79] was grown on soil under short day (10/14 hours light/dark) with 120 µmol photons.m^−2^.s^−1^ light at 23/19°C in controlled growth chambers (Conviron). Intact chloroplasts were purified from fully developed leaf rosettes. Chloroplasts were lysed and stroma and thylakoid membranes were collected principally as described in [80]. A low density membrane-enriched fraction was collected by high speed centrifugation (20 min at 120.000 *xg*) of stromal extracts. Tables S6A,B show the ratios of weighed SPC between stroma and thylakoid samples for some of the 156 proteins, which were identified in all nine experiments and provides a good indication of their subchloroplast localization.

### Protein analysis by Mass spectrometry

400 µg of thylakoid or stromal proteins were separated by SDS-PAGE (12% acrylamide) and stained with Coomassie Brilliant blue. Each gel lane was cut into 12 bands followed by reduction, alkylation, in-gel digestion with trypsin and peptides were extracted, as described in [81]. Peptide extracts were dried down and resuspended in 15–20 µl 5% formic acid for MS/MS analysis by either reverse phase nanoLC-ESI-QTOF (Micromass/Waters) or reverse phase nanoLC-ESI-LTQ-Orbitrap (Thermoelectron) and typically 6.4 µl were injected for each run.

The nanoLC-Q-TOF was interfaced with a CapLC system (Waters) and an autosampler from Waters. Analysis by nanoLC-Q-TOF was as follows: Used automated sample pickups, 6.4 µl peptide extracts were loaded at 15 µl.min^−1^ for 6 min on a guard column (LC Packings; MGU-30-C18PM), followed by elution and separation on a PepMap C18 reverse-phase nano column (LC Packings nan75-15-03-C18PM), using 90-min gradients with 95% water, 5% acetonitrile (ACN), 0.1% FA (solvent A), and 95% ACN, 5% water, 0.1% FA (solvent B) at a flow rate of 200 nl/min. A precolumn splitter was used to reduce the flow rate of 10 µl.min^−1^ to 200 nl.min^−1^. Each sample injection and analysis was followed by two blank injections using 60-min and 40-min gradients to prevent carry over from sample to sample.

The Q-TOF was operated in positive ion mode with sample cone voltage of 35 kV, capillary voltage of 3.3 kV and a source temperature of 90°C. The samples were run in data-dependent mode (DDA) where each full MS scan was followed by three consecutive MS/MS scans of the 3 most abundant peptide molecular ions (typically doubly and triply charged ions), which were selected consecutively for collision-induced dissociation (CID). The MS survey scans (m/z 350–1550) were carried out with a scan time of 1 second (s) and a interscan time of 0.08 s. MS/MS spectra were automatically acquired when the peak intensity rose above a threshold of 10 counts.s^−1^. Normalized collision energies for peptide fragmentation were set using the charge–state recognition files for 1+, 2+, 3+, 4+ peptide ions provided by Masslynx (Waters). For tandem MS acquisition we used a scan range from m/z 50–1550 with a scan time of 1.92 s and an interscan of 0.08 s and a dynamic exclusion window of 240 s. Argon was used as collision gas. All MS data from the Q-TOF were processed using Mascot Distiller (v2.0) and the resulting peak lists (in mgf files) were searched again against ATH1v6 database concatenated with a decoy where all the sequences were in reversed orientation using Mascot with a significance threshold of 0.01. For each of the peak lists two searches were performed: 1) tryptic search and 2) a semi-tryptic search with methionine oxidation set as a variable modification and carboxymethylation of cysteins as fixed modification and the maximum mass error tolerance for precursor and products ions at respectively 1.2 and 0.8 Da. Using in-house written filter the results both searches were combined in an excel spreadsheet. Only matches from tryptic search were considered in protein identifications. The Mascot output was automatically processed by in-house software (Qi Sun, B. Zybailov and K.J. Van Wijk; unpublished).

The LTQ-Obtrap was interfaced with an LC system (MS surveyor pumps from Thermoelectron) and an MicroAS autosampler (from Thermoelectron). Analysis by nanoLC-LTQ-Orbitrap was as follows: Used automated sample pickups, 6.4 µl peptide extracts were loaded at 20 µl.min^−1^ for 6 min on a guard column (LC Packings; MGU-30-C18PM), followed by elution and separation on a PepMap C18 reverse-phase nano column (LC Packings nan75-15-03-C18PM), using 90-min gradients with 95% water, 5% acetonitrile (ACN), 0.1% FA (solvent A), and 95% ACN, 5% water, 0.1% FA (solvent B) at a flow rate of 200 nl/min. A precolumn splitter was used to reduce the flow rate of 25 µl.min^−1^ to 200 nl.min^−1^. Each sample injection and analysis was followed by two blank injections using 60-min and 45-min gradients to prevent carry over from sample to sample. The acquisition cycle consisted of a survey FTMS scan at the highest resolving power (100,000), followed by 5 data-dependent MS/MS scans acquired in the LTQ. Dynamic exclusion was used with the following parameters: exclusion size 500, repeat count 2, repeat duration 30 s, exclusion time 180 s, exclusion window ±6 ppm. Target values were set at 5×10^5^ and 10^4^ for the survey and Tandem MS scans, respectively, and the maximum ion accumulation times were set at 200 ms in both cases. Acquired MS/MS data were searched with Mascot 2.2 using a significant threshold of 0.01. Preliminary search was conducted with broad precursor tolerance window set at ±30 ppm. Peptides with the ion scores above 45 were chosen as benchmarks for off-line recalibration. Recalibration was performed using a Perl script which adjusted precursor masses in the peak lists. The recalibrated peak lists were searched again against ATH1v6 database concatenated with a decoy where all the sequences were in reversed orientation. For each of the peak lists three searches were performed: 1) *tryptic* search with precursor ion tolerance window set at ±6 ppm, 2) *error-tolerant* search with precursor ion tolerance window set at ±3 ppm, 3) *semi-tryptic* search with acetylation of peptide N-terminus set as a variable modification. For all of the three types of searches first and second ^13^C peaks were considered as precursors without widening of the precursor ion tolerance, using the corresponding Mascot 2.2 feature. Additionally, in all of the three searches methionine oxidation was set as a variable modification. Using in-house written filter the results of the three searches were combined in an excel spreadsheet. The ion scores threshold were set to 33, which yielded final peptide false positive rate below 1%. Additionally, for proteins represented only by one unique peptide, mass accuracy on the precursor ion was required to be within ±3 ppm. Only matches from tryptic search were considered in protein identifications.

### Calculation of protein abundance

For each of the proteins identified in stromal preparations P1–P3, total number of spectral counts (SPC) and number of unique SPC was extracted from the Mascot 2.2 output using in-house written filters. In cases where two or more homologous proteins were identified with shared and unique peptides, the number of spectra from shared peptides assigned to each protein was determined based on the ratios of spectra derived from the unique peptides that identified each protein, similar to [43], using formula:
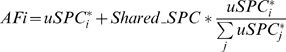
where 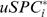 is the initial number of unique SPC for *i*-th protein the group, *AFi* is the abundance factor used for quantification, Shared_SPC – number of total shared SPC within the group, and 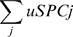 is the sum of all unique SPC within the group. In cases of ambiguous groups (no unique SPC), as well as in the cases of protein subsets *AF* was set equal to *Shared_SPC*, with the whole ambiguous group treated as one protein.

To obtain measure of protein abundance, normalization formula
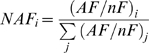



was used, with normalization factor, nF, derived from mature protein sequence (transit peptide(s) removed) being either length in number of aa residues or, alternatively, number of fully tryptic peptides in the mass range of 700 to 3500 Da.

### Statistical analysis of reproducibility by G-test

G-test was performed across three technical and across three biological replicates of chloroplast stroma preparations as described in [17], [40] and in [39]. Briefly, for each protein across *m* conditions tested, the G-statistic is calculated and then compared to the Chi-square distribution with *m*−1 degrees of freedom, resulting in a p-value. Finally, a correction for multiple testing is applied to the p-values, and proteins with significant changes in abundance are determined. The higher the number of proteins with significant changes, the poorer is reproducibility.

### Analysis of transit peptides and cleavage sites

Sequence logos were created using the web server at http://weblogo.berkeley.edu/. The plots of binned frequencies were obtained by counting the aa frequencies at the two N-terminal positions and 5 positions (2 in cTP, 3 in mature protein) around the predicted cTP cleavage site separately (i.e., one bin for each of these positions), and cutting up the remaining internal part of the cTP in 20 bins. In total 27 bins. This is a simplistic way to investigate regional properties of all cTPs regardless of their length differences. Multiple sequence alignments of the cTPs using Clustalw with standard scoring matrices and parameter settings were uninformative.

### PPDB structure and content

The Mascot output files were automatically processed by in-house software (Qi Sun, B. Zybailov and K.J. Van Wijk, unpublished) and a number of output parameters (accession number, instrument type, experimental ambiguity, Mowse score, number of matching peptides, number of matched MS/MS spectra (queries), number of unique queries, highest peptide score, highest peptide error (in ppm), lowest absolute error (ppm), sequence coverage, tryptic, semi-tryptic peptide and modified peptide sequences) were uploaded into the PPDB.

The construction of the PPDB (http://ppdb.tc.cornell.edu/) was originally described in [36]. Since its inception in 2004, the PPDB interface was improved and we renamed the data base from Plastid Proteome DB to Plant Proteome DB to better reflect the content. The ‘backbone’ of the PPDB are all protein-encoding accessions (currently release 6.0 of ATH1.pep) with a theoretical analysis (predicted localization and physical properties of precursor and processed proteins) of all *Arabidopsis* entries. Also, more detailed curated information about the MS based identification can now be accessed; this will allow the user to better evaluate the strength of protein identification. Mascot and ion scores, mass accuracies, matched aa sequences, number of matching peptides and highest peptide score for each identification and other MS based data are listed. The MapMan Bin system [45] is used for functional assignment and all assignments for identified proteins were verified manually.

To determine potential non-chloroplast contaminants in this filtered dataset, we used an extensive cross-correlation to more than fifty published proteomics papers on *Arabidopsis* subcellular fractions, as well as information extracted from TAIR, from SUBA [53], and other literature. The complete list of published proteomics papers and each of their accessions can be downloaded from PPDB (http://ppdb.tc.cornell.edu/searchpub.aspx). As a rule of thumb, subcellular localization by GFP/YFP and western blots was considered strong evidence, although we noted that there are several examples of incorrect subcellular localization assignment based on GFP/YFP, as discussed in [12]. Identification in published proteomics studies was sometimes difficult to judge since information about the ‘strength’ of MS based identification was not easily accessible.

Chloroplast proteins are among the most abundant cellular proteins, and contaminations from the chloroplast are often observed in proteome analyses of other organelles. However, if proteins with lower abundance ranks were identified in multiple non-plastid proteomics studies from the same subcellular fraction (*e.g*. cell wall or plasma membrane), they were considered contaminants. To avoid circular arguments when evaluating TargetP (below), predicted chloroplast localization was not considered in assigning proteins to the chloroplast.

In addition, all protein accessions can be directly cross-referenced against identified proteins from more than 50 published proteomics papers (from *A. thaliana* and other members of the *Brassicae* family), as well as the subcellular localization in GO (experimental) from TAIR, localization data from SUBA (http://www.suba.bcs.uwa.edu.au) and best matching homologues in maize and rice; Many of these data types can be directly exported from the PPDB as excel files.

## Supporting Information

Text S1 Analysis of technical and biological variation.
(0.03 MB DOC)

Table S1 Chloroplast proteome analysis by LTQ, identifying 1258 non-ambiguous proteins and 22 ambiguous protein groups.
(0.33 MB XLS)

Table S2 Abundance information of 946 stroma-enriched proteins determined by mass spectrometry and correlation to gel-based quantification.
(0.40 MB XLS)

Table S3 Analysis by Q-TOF of seven independent stromal preparations identifying 623 non-redundant proteins (counting only 1 gene model) with 1 or more unique peptides.
(0.10 MB XLS)

Table S4 Identified and annotated proteins from filtered experimental MS/MS experiments and additional 200 annotated chloroplast proteins from literature.
(0.26 MB XLS)

Table S5 Sequences and mass spectrometry details of N-terminal acetylated nuclear-encoded proteins.
(0.02 MB XLS)

Table S6 Assessing quality of sub-organelle enrichment by comparing protein levels in Stroma and Thylakoids. (A) Top 27 proteins over-represented in stroma protein preparations. (B) Top 27 proteins over-represented in thylakoid preparat
(0.57 MB XLS)
