# Supplementary material for: Sorting Signals, N-Terminal Modifications and Abundance of the Chloroplast Proteome
Source: PLoS One. 2008 Apr 23;3(4):e1994. doi: 10.1371/journal.pone.0001994 (PMC2291561; doi:10.1371/journal.pone.0001994)
Supplement: Text S1 — Analysis of technical and biological variation. (0.03 MB DOC) [file pone.0001994.s001.doc]

***Supplemental Text***

***Analysis of technical and biological variation***

Technical replicates R1, R2, R3 of the stroma from the first chloroplast preparation (P1) had 7289, 7433 and 5433 total unique number of spectral counts (SPC) (Figure 1A and Table 1) R3 was run as the residual of the sample P1, explaining the 25-30% lower number of counts and identified proteins, as compared to R1 and R2. The overlap in identified proteins between R1 and R2 was 82%. The similarity between R1 and R2 in terms of counts, total protein Mowse score and identified proteins, allows assessing technical variations associated only with the chromatography and mass-spectrometric acquisition method. The chromatographic reproducibility, as measured by variation in retention times and heights of major peaks was good (respectively, on average within 8 seconds and within 9% coefficient of variation) suggesting that the main source of technical variation is the MS/MS acquisition. The most likely reason for this variation is ‘undersampling’, which is the case when not every MS peak is chosen for MS/MS fragmentation (Elias et al., 2005). Inspection of the identified proteins shows that low-abundant chloroplast proteins and non-chloroplast contaminants were most frequently undersampled, and as a consequence, they are mostly present in the non-overlapping areas of the Venn diagrams in Figure 1. This enrichment of contaminants is also reflected by the lower percentage of predicted cTPs in the non-overlapping areas.

To further assess the reproducibility between technical and biological replicates, we then carried out a G-test of independence of the SPC (Sokal, 1995) (Old et al., 2005; Zhang et al., 2006). We applied the G-test to stromal technical replicates R1 and R2 from chloroplast P1 and found only one protein out of 720 with significant (p<0.01) difference in unique SPC. This shows that the technical variation resulting from on-line chromatography and MS analysis is very small. The average coefficient of variation (cv) of the SPC between R1 and R2 was 25%. When we applied the G-test to the SPC of the three stromal biological replicates, 113 out of 913 proteins (~12%) showed significant changes in unique SPC. This likely reflects a combination of variation between biological samples, chloroplast isolation and fractionation. Manual inspection of these 113 proteins shows that they contain indeed ~25% non-stromal proteins associated with the thylakoid and envelope, and a few non-chloroplast contaminants. The remaining ~75% represent stromal proteins and showed an overrepresentation of the most abundant proteins and the least abundant proteins, observed only once or twice. The average cv of SPC, normalized by the total number of SPC between the three biological stromal replicates was 52%, considering only proteins identified in every preparation. Given that SPC for identified proteins span 4 orders of magnitude, this cv is very low.
